# Effects of intestinal lymphatic ligation on intestinal immunity in rats with severe acute pancreatitis

**DOI:** 10.1002/2211-5463.13115

**Published:** 2021-02-28

**Authors:** Yuanqi Liu, Li Chen, Lulu Wang, Yuxia Xiong

**Affiliations:** ^1^ College of Comprehensive Health Management Xihua University Chengdu China; ^2^ Department of Pharmacy The Affiliated T.C.M. Hospital of Southwest Medical University Luzhou China; ^3^ College of Pharmacy Southwest Medical University Luzhou China

**Keywords:** intestine, ligation, mesenteric lymph duct, sever acute pancreatitis

## Abstract

Severe acute pancreatitis (SAP) is one of the most common diseases of the gastrointestinal tract, characterized by a complicated pathogenesis, multiple organ failure, and high mortality. The primary aim of the present study was to observe the effect of intestinal lymphatic ligation on intestinal injury and modification in rats with SAP. Male Sprague‐Dawley (SD) rats were randomly divided into: (a) Saline group (SO); (b) SAP group; and (c) SAP + ligation group. We evaluated the effect of mesenteric lymphatic duct ligation on the pancreas and intestine tissue by HE. The histopathology of the pancreas in SAP + ligation rats was alleviated slightly compared with SAP rats, but aggravated in the intestine of SAP + ligation rats. Treatment of mesenteric lymphatic duct ligation resulted in an increase in the levels of tumor necrosis factor (TNF)‐α, interleukin (IL)‐1β, and myeloperoxidase compared with the small intestinal tissues of SAP rats. In addition, the expression of nucleotide‐binding oligomerization domain‐like receptors 3, apoptosis‐associated speck‐like protein containing a caspase recruitment domain (CARD) (ASC), and caspase‐1 in the intestine were higher in the SAP + ligation group. The ratio of Th1/Th2 and regulatory T cells (Tregs) in the mesenteric lymph nodes of the SAP group was lower than those in the SAP + ligation group. The present results indicated that ligation of the mesenteric lymph duct can effectively prevent intestinal inflammatory mediators entering the body through the mesenteric lymph duct, but these mediators assembled in the intestine where they induced an excessive immune response and intestinal injury during SAP.

## Graphical abstract

In severe acute pancreatitis (SAP) rats, blocking the intestinal lymph pathway led to a large number of inflammatory cytokines in the intestinal tract, which increased intestinal tract damage. The present study may help elucidate the role of the intestinal lymphatic system in SAP from a novel perspective and provide evidence that the gut lymphatic system may be a potential target for drug treatment.

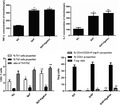

**Abbreviations**
(TNF)‐α: tumor necrosis factor
ASC: apoptosis‐associated speck‐like protein containing a caspase recruitment domain
GALT: gut‐associated lymphoid tissue
IL‐1β: interleukin‐1β
LPL: lamina propria lymphocyte
MLNs: intestine and mesenteric lymph nodes
MPO: myeloperoxidase
NLRP3: nucleotide‐binding oligomerization domain‐like receptors 3
SAP: severe acute pancreatitis
SIgA: secretory immunoglobulin A
Treg: regulatory T

Severe acute pancreatitis (SAP) is one of the most common diseases of the gastrointestinal tract, characterized by a complicated pathogenesis, multiple organ failure, and high mortality [1, 2]. The pathogenesis of SAP is a complex pathophysiological process, which has not been fully elucidated. It has been suggested that the intestinal tract is the central organ of the stress response and involved in multiple organ dysfunction syndrome (MODS) secondary to SAP.

It has been recognized that bacteria and/or endotoxin translocation from the intestinal lymphatic duct, but not the vein, into the blood circulation is associated with severe complications, such as SAP, systemic inflammatory response syndrome, sepsis, MODS, and multiple organ failure [3]. The intestinal lymphatic system plays an important role in SAP. The intestinal lymphatic system consists of the gut‐associated lymphoid tissue (GALT) and intestinal lymph duct. GALT may facilitate the release of leukocytes from these tissues into the lymphatic circulation [4, 5]. Additionally, the intestinal lymph duct is the anatomical pathway that connects pulmonary circulation and the intestines [6, 7]. Sensitized T and B lymphocytes assemble into the pulmonary and blood circulation via the mesenteric lymph duct and thoracic duct, and become effector cells [8, 9]. Mucosal addressin cell adhesion molecule‐1 plays a role in the transfer of mucosal lymphocytes into the effector cells. Besides bacteria, endotoxin, cytokines, and other immune responses produced by stimuli can also be transferred to the lungs and blood via the intestinal lymphatic system [10].

The gastrointestinal system is a common entry point for pathogenic microbes to access the internal environment of the body [11]. The intestine harbors high numbers of commensal bacteria [12]. Hammer *et al*. [13] demonstrated that, at the histological level, the small and large intestines contain a barrier of mucous and epithelial cells that block the translocation of bacteria in the lumen to sites in the body beyond the intestines. The intestinal flora and its products play a crucial role in the induction of the intestinal lymphatic system of the innate immune response and adaptive immune response by increasing Immunoglobulin A (IgA) production and mucin expression [14, 15]. IgA is a key molecule that limits microbiota adherence and access to the intestinal epithelial surface [16]. In the gut, regulatory T cells (Tregs), characterized by the expression of the forkhead family transcription factor, and forkhead box P3 (FOXP3) have also been observed to be a primary mediator that maintain immune homeostasis [17]. FOXP3^+^ Tregs can convert into T helper (Th)1, Th17, and follicular Th cells in the intestine, especially during inflammation [18, 19, 20, 21].

However, no study has fully elucidated a target in the intestinal lymphatic system for the treatment of SAP. Therefore, to explore the action and underlying mechanisms of mesenteric lymph duct ligation in SAP is of high importance. Our previous work on SAP indicated that mesenteric lymph duct ligation can alleviate lung, kidney, and liver injury by preventing the intestinal inflammatory factor shift in rats, thus reducing systemic endotoxins, tumor necrosis factor (TNF)‐α, interleukin (IL)‐1β, and myeloperoxidase (MPO) in lung, kidney, and liver tissue [22, 23]. In contrast, the damage of the small intestine was slightly worse than that of the model group after ligation of the intestinal lymphatic vessels. The aforementioned results showed that the intestinal lymphatic pathway was one of the more important pathways for inflammatory transfer to mediate remote organ injury. The result differed greatly from the traditional hypothesis that damage factors entered into the blood circulation primarily via portal vein migration.

We hypothesized that intestinal lymphatic ligation may play a role in systemic inflammatory response induced by pancreatitis by regulating the intestinal lymphatic system immune response. If the gut lymphatic system was the primary route of production and translocation of intestinal damage factors, then inflammatory and the immune response effector cells were retained in the gut and resulted in a more robust immune response in the gut. The results of the present study may help elucidate the role of the intestinal lymphatic system in SAP from a novel perspective and provided evidence that the gut lymphatic system may be a potential target for drug treatment.

## Materials and methods

### Experimental animal

Clean grade, healthy male Sprague‐Dawley (SD) rats weighing 200 ± 20 g were purchased from the Laboratory Animal Center, Southwest Medical University [SCXK (chuan) 2013‐24]. Rats were maintained in a temperature‐controlled environment at 24 °C with a 12‐h light/dark cycle and were provided with drinking water and feed *ad libitum*. All efforts were taken to minimize the suffering of animals used. During housing, animals were monitored twice daily for health status. No adverse events were observed. The rats were acclimatized in the laboratory conditions for a period of 1 week before being used in the experiment. Eighteen rats were randomly divided into: (a) Saline group (SO; *n* = 6); (b) SAP group (*n* = 6); and (c) SAP + ligation group (*n* = 6). 3.5% sterile sodium taurocholate solution (STC, 1 mL·kg^−1^; St. Louis, MO, USA) was injected into the biliopancreatic duct of rats to prepared for the SAP group and SAP + ligation group. In addition, the mesenteric lymphatic ducts of the SAP + ligation group, which ran along the superior mesenteric artery, were separated and were only threaded with silk below, and then, the abdomen was closed.

After 24 h, the rats were successfully prepared. The rats were anesthetized with an intraperitoneal injection of 50 mg·kg^−1^ of 2.0% pentobarbital sodium (Sigma, St. Louis, MO, USA). Rats were euthanized by cervical dislocation. The tissue specimens of rat ileum (~ 200 mg), pancreas, and MLN were collected. MLN was prepared for single cell suspension. A part of intestinal tissues was preserved in −80 °C refrigerator for the preparation of tissue homogenate for ELISA. Another part of tissues was fixed in 10% paraformaldehyde for 4 h, then washed for 24 h with tap water, and then observed the pathological changes and immune index. The study was approved by the Southwest Medical University, and the experimental procedures were in accordance with the ‘Guidelines for the Protection and Use of Laboratory Animals’.

### Hematoxylin and eosin staining

Pancreas and terminal ileum specimens were stained with hematoxylin and eosin (HE). Thin slices of pancreas and intestine tissues for all groups were fixed in 4% formaldehyde solution (pH 7.0) for periods not exceeding 24 h. The tissues were processed routinely for paraffin embedding, and 4 µm‐thick sections were cut and placed on glass slides. Tissue samples were stained with HE and observed by the microscope (Olympus, model CX31, Shinjuku‐Ku, Japan). The morphologic changes in the pancreas, including pancreatic edema, acinar cell necrosis, adipose necrosis, hemorrhage, and inflammation, were scored according to the criteria of Schmidt (Schmidt's score: 0–4, edema, neutrophil infiltration, necrosis, and hemorrhage, resp.) [24]. The pathological damage of the ileum was graded according to Chiu's standard. Chiu's score was graded as follows: Grade 0: normal mucosa; Grade 1: formation of subepithelial detachments at the tip of the villi with capillary congestion; Grade 2: subepithelial detachments exert a moderate amount of upward push on the mucosal epithelium; Grade 3: large subepithelial detachments exert a massive amount of upward push on the mucosal epithelium along the villi and a few denuded villus tips are observed; Grade 4: the villi are denuded to the level of lamina propria and dilated capillaries; Grade 5: presence of ulceration, disintegration of lamina propria, and hemorrhage [25].

### Enzyme‐linked immunosorbent assay

An ELISA (Long Beach, CA, USA) was performed to determine the levels of the pro‐inflammatory cytokines TNF‐α and IL‐1 using commercially available kits from R&D Systems (Minneapolis, MA, USA). Optical density was measured on an ELISA plate scanner (Bio‐Rad 680, Hercules, CA, USA) at 490 nm. The results were expressed as picograms of TNF‐α or IL‐1 per milliliter of intestine tissue.

### MPO assay

Frozen intestine tissues were homogenized and processed for measurement of MPO activity. The MPO Activity Assay kit (Nanjing Jiancheng Bioengineering Institute, Nanjing, China) was used for MPO determination according to manufacturer instructions.

### Analysis of NLRP3 and ASC by immunofluorescence

Samples were baked in the oven at 65 °C for 2 h and dewaxed, repeatedly with xylene I, II, III, for 10 min and hydrated sequentially in 100% EtOH, 95% EtOH, 80% EtOH, respectively, for 5 min, 70% EtOH for 2 min, and then distilled water for 2 min. After hydration, washed three times in PBS for 5 min. Dropped 3% H_2_O_2_ of deionized water on the sample and incubated for 10 min to block endogenous peroxidase, and then washed three times with PBS for 5 min. Antigen repair: added citric acid repair solution (PH 6.0), heated to 95 °C, sliced, cooked for 20 min. After stopping heating, cooled naturally to room temperature and washed three times with PBS for 5 min. Samples were absorbed by filter paper, added 0.3% TritonX‐100 for 10 min, and washed three times with PBS for 5 min. Blocking antigen: dried water with filter paper and added sealing liquid (5% FBS) for 30 min. Erased sealing liquid with filter paper and do not wash. Added primary antibody (Delaware Ave Santa Cruz, Santa Cruz, CA, USA; 1 : 50), put into the wet box, placed in the refrigerator at 4 °C, and incubated overnight. After rewarming for 1 h, washed three times with PBS for 5 min, dried residue PBS, added second antibody (ZSGB, Beijing, China; 1 : 100) with fluorescence, placed in the wet box at 37 °C, and incubated for 30 min avoiding meeting up. Then washed three times with PBS for 5 min and wiped out the PBS to take photographs [26].

### Western blotting

Equal amounts of total protein were loaded onto 12% SDS/PAGE at 80 V for 80 min, electro transferred to polyvinylidene difluoride membranes by the wet transfer method, and blocked in 5% BSA at 4 °C overnight. Subsequently, the membranes were incubated with an anti‐β‐actin antibody (Santa Cruz, CA, USA; 1 : 1000) and anti‐nucleotide‐binding oligomerization domain‐like receptors 3 (NLRP3) antibody (Santa Cruz, CA, USA; 1 : 1000) at room temperature for 2 h. After washing with TBST, the membranes were incubated with secondary goat anti‐mouse IgG antibody (Santa Cruz, CA, USA; 1 : 500) or goat anti‐rabbit IgG antibody (Santa Cruz, CA, USA; 1 : 500) at room temperature for 1 h. Equal loading of protein in each lane was verified by reblotting the membrane with an anti‐β‐actin antibody (ZSGB, Beijing, China). Then, proteins were detected by chemiluminescence reagent. Protein band density was quantified using Bio‐Rad quantity one v4.62.

### Analysis of Caspase‐1 and SIgA by immunohistochemistry

Samples were baked in the oven at 65 °C for 2 h and dewaxed repectly with xylene I, II, III, for 10 min and hydrated sequentially in 100% EtOH, 95% EtOH, 80% EtOH, respectively, for 5 min, 70% EtOH for 2 min, and then distilled water for 2 min. After hydration, washed three times in PBS for 5 min. Antigen repair: added citric acid repair solution (PH 6.0), heated to 95 °C, sliced, cooked for 20 min. Dropped 3% H_2_O_2_ of deionized water on the sample and incubated for 10 min to block endogenous peroxidase, and then washed 3 times with PBS for 5 min. Added primary antibody (Delaware Ave Santa Cruz; 1 : 50), put into the wet box, placed in the refrigerator at 4 °C, and incubated overnight. After rewarming for 1 h, washed three times with PBS for 5 min, dried residue PBS, added second antibody (ZSGB; 1 : 100) with fluorescence, placed in the wet box at 37 °C, and incubated for 30 min avoiding meeting up. Applicated DAB solution for coloration for 5 min and washed by distilled water. Redyeing: nuclear staining with hematoxylin for 5 min, washed with tap water, and observed under the microscope. If hypochromasia, stained with hematoxylin once again. If deep, redifferentiated with hydrochloric acid and stained with hematoxylin. Hydrated sequentially in 100% EtOH, 95% EtOH, 80% EtOH, respectively, for 5 min. Mounted with neutral balata.

### Cell isolation

Part of MLN was taken and placed in a sterile plate containing precooled (4 °C) PBS, and the membrane was removed. Then, it was transferred to the 400 mesh sieve and cut into pieces by ophthalmology scissors. The MLN was gently ground with the inner core of a 2‐mL syringe to remove the cell mass and prepare single cell suspension the MLN. Centrifugation was performed at 1000 r.p.m. for 5 min. The supernatant was removed and repeated once. Cells of MLN were resuspended in complete medium, and the concentration was adjusted to 10^6^/mL by cell counting plate for flow cytometry.

### Analysis of Th1/Th2 ratio by flow cytometry

The three groups of cell suspension were added into the sterile 6‐well cell culture plate. Each hole was added with 2 μL monensin by pipette avoiding air bubbles. The plate was put into cell culture box for 5 h. After the collection of cells and centrifugation (350 ***g***) for 5 min, removed the supernatant. Two millilitre PBS were added into the tube, centrifugated (100 ***g***) for 5 min, and removed the supernatant, and respectively, the MLN cells were suspended into the EP tube. Five microliter the corresponding surface marker antibody CD4 (BioLegend, San Diego, CA, USA) was added to each tube and incubated at room temperature for 15 min in the dark. The MLN cells were suspended with PBS, centrifugated (350 ***g***) for 5 min, and removed the supernatant. Fixed cell with 0.5 mL fixed liquid, incubated at room temperature for 20 min in the dark, centrifugated (350 ***g***) for 5 min, and removed the supernatant. Added 500 μL PBS to suspend cells and restored overnight at 4 °C in the dark. Centrifugated (350 ***g***) for 5 min and removed the supernatant. Resuspended MLN cells with 1 mL membrane rupture, centrifugated (350 ***g***) for 5 min, and removed the supernatant repeatedly. Resuspended MLN cells with 100 μL membrane rupture, added 5 μL IL‐4‐FITC (BioLegend, CA, USA) and 20 μL IFN‐γ‐PC5 (BioLegend) into the tube, and incubated at room temperature for 20 min in the dark. Washed MLN cells two times with PBS centrifugated (350 ***g***) for 5 min and removed the supernatant [27]. Took 0.3 mL PBS suspension cell to analyze by FCM.

### Analysis of Treg by flow cytometry

The MLN cells were suspended into the EP tube. Five microlitre the corresponding surface marker CD4‐FITC (BioLegend) and CD25‐PE (BioLegend) was added to each tube and incubated at room temperature for 20 min in the dark. The MLN cells were suspended with PBS, centrifugated (350 ***g***) for 5 min, and removed the supernatant. Fixed cell with 1 mL FOXP3 fixed liquid (BioLegend), incubated at room temperature for 20 min in the dark, centrifugated (350 ***g***) for 5 min, and removed the supernatant. Added 2 mL PBS to suspend cells and restored overnight at 4 °C in the dark. Centrifugated (250 ***g***) for 5 min and removed the supernatant. Resuspended MLN cells with 1 mL FOXP3 membrane rupture (BioLegend), incubated at room temperature for 20 min in the dark, and removed the supernatant repeatedly. Added 2 mL PBS to suspend cells and restored overnight at 4 °C in the dark. Centrifugated (250 ***g***) for 5 min and removed the supernatant. Resuspended MLN cells with 100 μL FOXP3 membrane rupture. Centrifugated (250 ***g***) for 5 min and removed the supernatant. MLN cells were resuspended with 1ml FOXP3 membrane rupture, incubated at room temperature for 20 min in the dark, centrifugated (250 ***g***) for 5 min, and removed the supernatant. MLN cells were resuspended with 100 μL FOXP3 membrane rupture. Added 5 μL FOXP3‐APC (BioLegend) into the tube, and incubated at room temperature for 30 min in the dark. Washed MLN cells two times with 2 mL PBS, centrifugated (250 ***g***) for 5 min, and removed the supernatant. Took 0.3 mL PBS suspension cell to analyze by FCM. Gated strategy with CD4^+^ T cells. Lymphocytes were selected on the FSC/SSC scatter plot, and then, CD4 ^+^ lymphocytes were selected from the lymphocyte gate. In the above cells, CD25^+^ FOXP3^+^ cells were selected as Treg.

### Statistical analysis

Data were expressed as mean ± SD and analyzed by one‐way ANOVA followed by LSD and Dunnett's T3 using spss version 14 software (Chicago, IL, USA). *P* < 0.05 was considered as statistical significance.

## Results

### Pathological changes in the pancreas

**Fig. 1:**
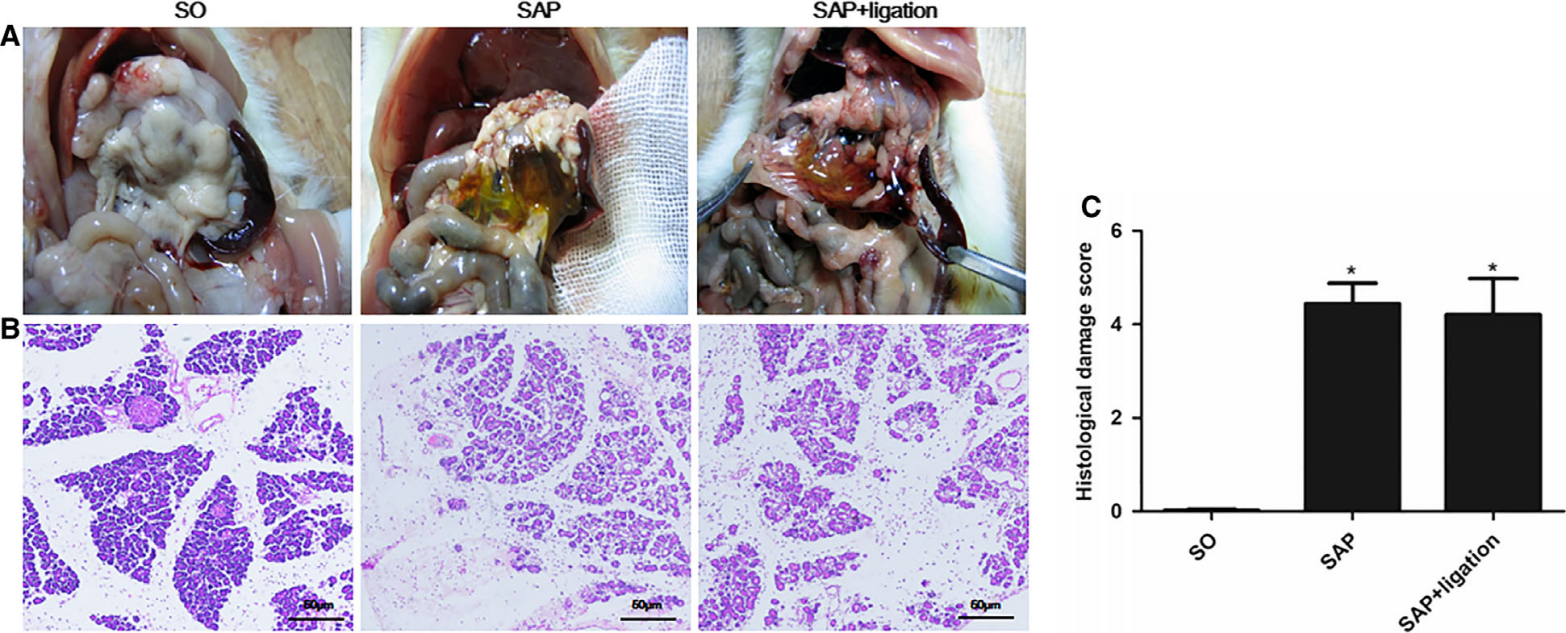
Change in pathological tissue of pancreas of rats and histological score of the pancreas in each group. (A) Pancreatic tissue (B) H&E stain of pancreatic tissue. (C) Histological score. The pancreatic sections were stained by H&E stain and observed under a microscopy (200× magnification). Scale bars = 50 µm. The data were presented as mean ± SD. There were six rats in each group (*n* = 6). *P* values were derived by LSD (L) test and Dunnett's T3 test.*P < 0.05 vs SO group.

To investigate the effect of mesenteric lymphatic duct ligation on pancreas of SAP rats, we observed histopathology of pancreas. Under light microscope, the structure of the pancreas in the SO group was mostly normal. SAP group showed congestion, edema, several spot hemorrhage, and inflammatory cell infiltration of pancreatic tissue. The inflammation was relieved slightly in the SAP + ligation group (Fig. 1). But there was no significant difference. The results indicated that there was no direct protective effect on pancreatic injury with mesenteric lymphatic duct ligation.

### Pathological changes in the intestinal mucosa

**Fig. 2:**
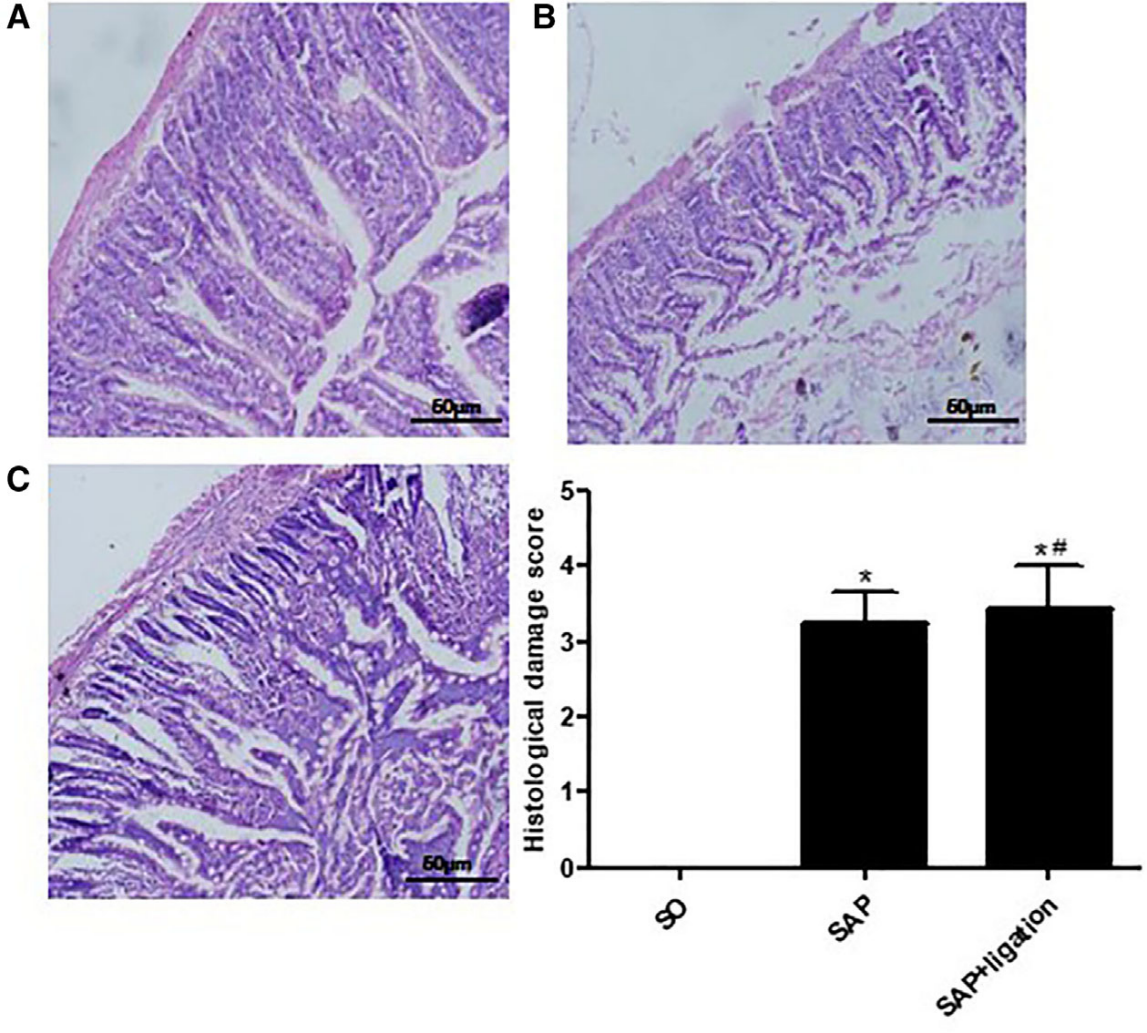
Change in pathological tissue of small intestine of rats and histological score of the intestine in each group. (A) SO (B) SAP (C) SAP + ligation. The intestinal sections were stained by H&E stain and observed under a microscopy (200× magnification). Scale bars = 50 µm. The data were presented as mean ± SD. There were six rats in each group (*n* = 6). *P* values were derived by LSD (L) test and Dunnett's T3 test. * *P* < 0.05 vs SO group; ^#^ *P* < 0.05 vs SAP group.

To investigate the effect of mesenteric lymphatic duct ligation on intestine of SAP rats, we observed histopathology of intestine. Under light microscope, the structure of the small intestinal mucosa in the SO group was mostly normal. After induction of SAP, the small intestine wall was characterized by congestion, edema, damage to the villi, epithelial degeneration, necrosis, and shedding. The lamina propria mucosa was separated. Dilation of blood vessels, hemorrhage, and inflammatory cell infiltration were observed in the submucosa. Conversely, in the SAP + ligation group, fusion and defect of intestinal villi were worse compared with the SAP group (Fig. 2). The degree of intestinal pathological injury in the SAP group and SAP + ligation group was significantly higher than those of the SO group. The SAP + ligation group was more serious than the SAP group (*P* < 0.05). The results indicated that intestinal inflammation was more serious in the SAP + ligation rats. Ligation of mesenteric lymph duct may prevent the intestinal damage factor from entering the blood circulation through the mesenteric lymphatic pathway, and stay in the intestinal tract. The inflammatory reaction remained in the intestine.

### Levels of TNF‐α, IL‐1, and MPO in intestinal tissue homogenate

**Fig. 3:**
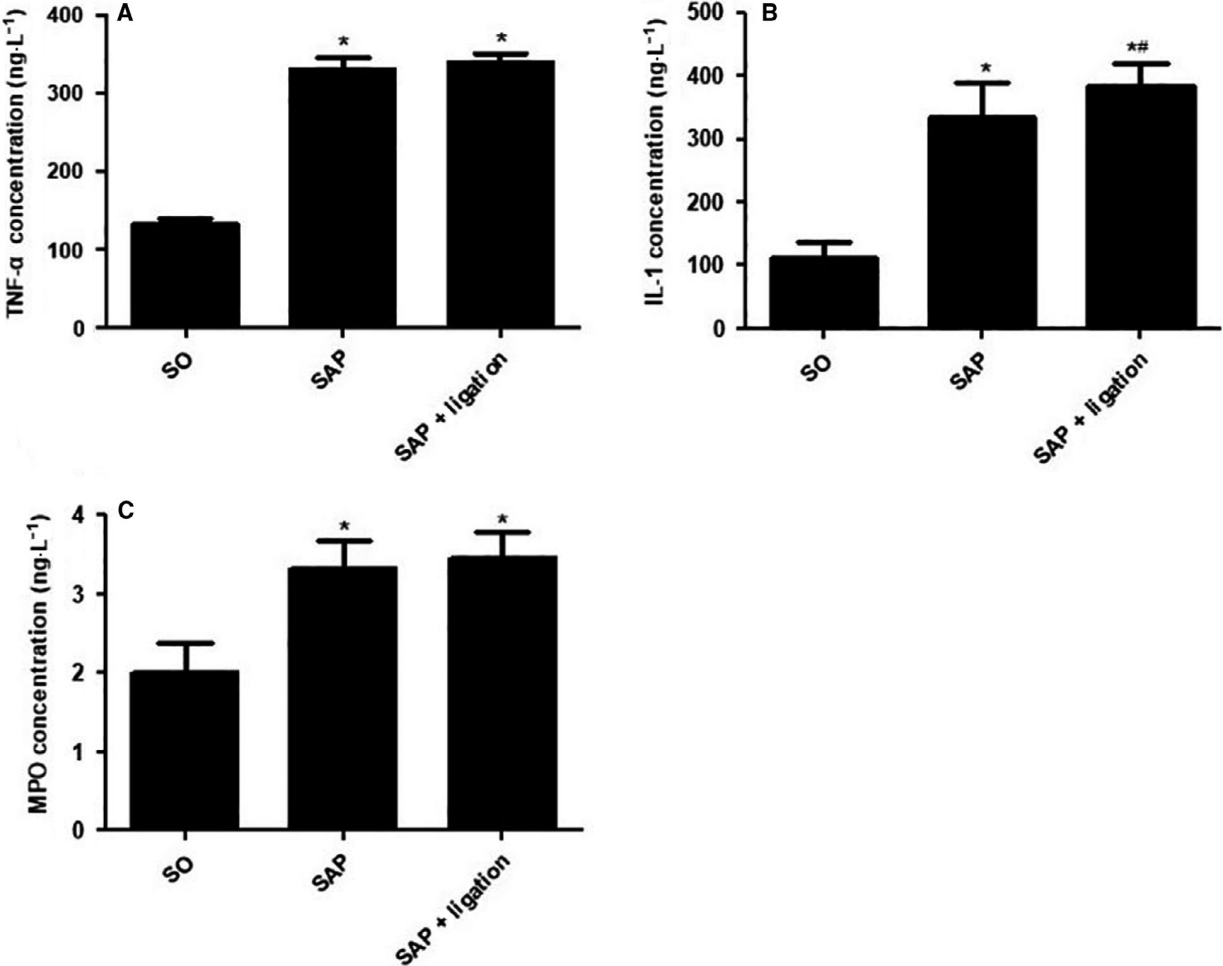
The levels of TNF‐α, IL‐1, and MPO in small intestinal tissues in rats. (A) TNF‐α (B) IL‐1 (C) MPO. There were six rats used for each experimental group (*n* = 6) and expressed as mean ± SD. *P* values were derived by LSD (L) test and Dunnett's T3 test.*P < 0.05 vs SO group; ^#^ *P* < 0.05 vs SAP group.

The levels of TNF‐α, IL‐1, and MPO in the SAP group were notably higher than those in the SO group (*P* < 0.05). The level of IL‐1 in the SAP + ligation group was significantly higher than that in the SAP group (*P* < 0.05). There were no significant differences in TNF‐α and MPO between the SAP group and SAP + ligation group (Fig. 3). The results indicated that intestinal lymphatic ligation may aggravate intestinal injury.

### Expression of NLRP3 and ASC in the small intestine and MLN of rats

**Fig. 4:**
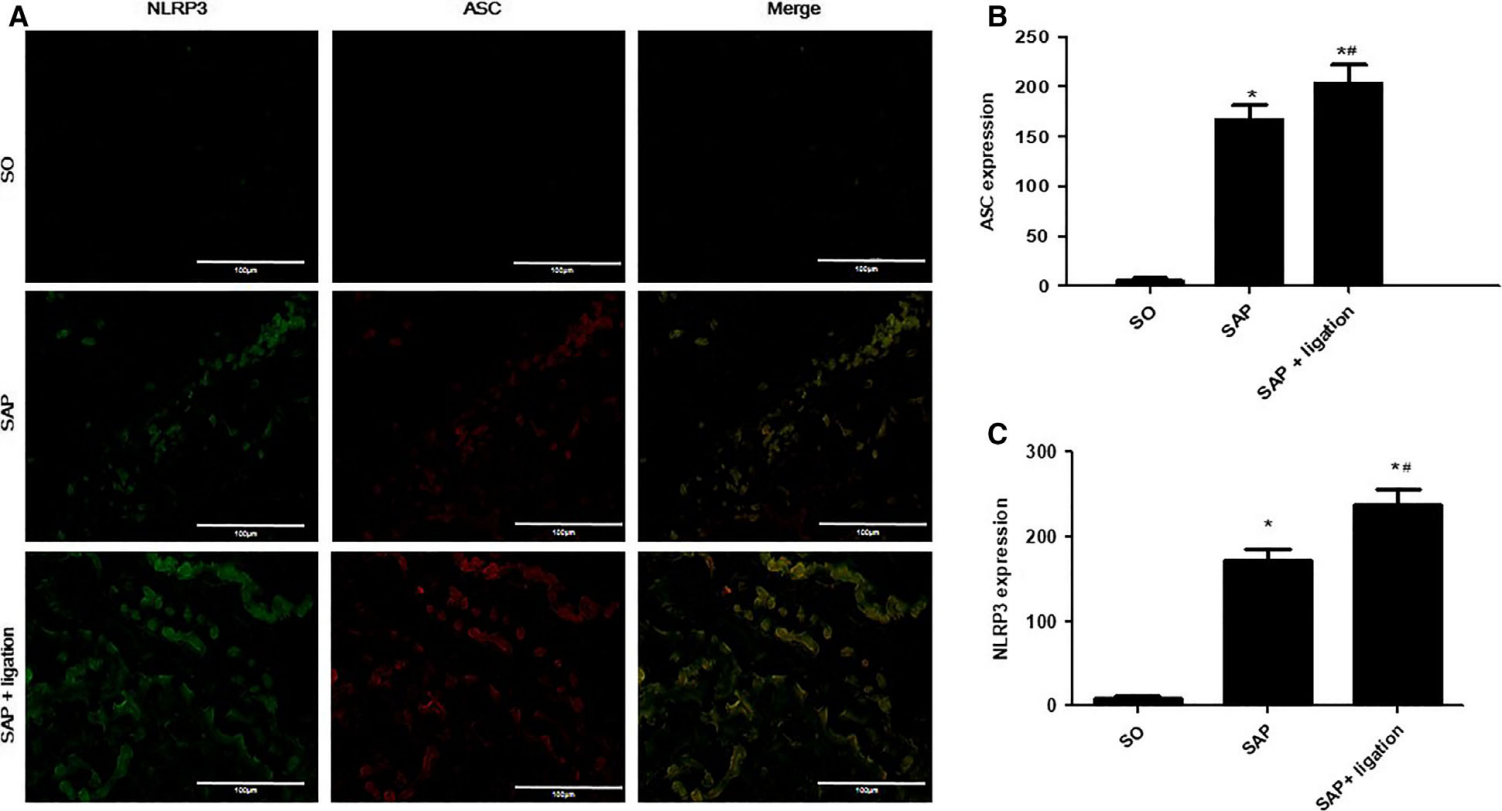
Effects of intestinal lymphatic ligation on the expressions of NLRP3 and ASC in the small intestinal tissues in rats. The representative images of the uptake of NLRP3 and ASC in the small intestinal tissues of rats (A) and summary results of bar graphs for each group (B and C). NLRP3 and ASC were determined by confocal laser scanning microscopy (400× magnification), positive expression of NLRP3 presented as green fluorescence, while positive expression of ASC presented as red fluorescence. The counts of positive expressions of ASC and NLRP3, the fluorescence expressions were counted at least 10 fields for each slide. Scale bars = 100 µm. There were six rats used for each experimental group (*n* = 6) and expressed as mean ± SD. *P* values were derived by LSD (L) test and Dunnett's T3 test. * *P* < 0.05 vs SO group; ^#^ *P* < 0.05 vs SAP group.

**Fig. 5:**
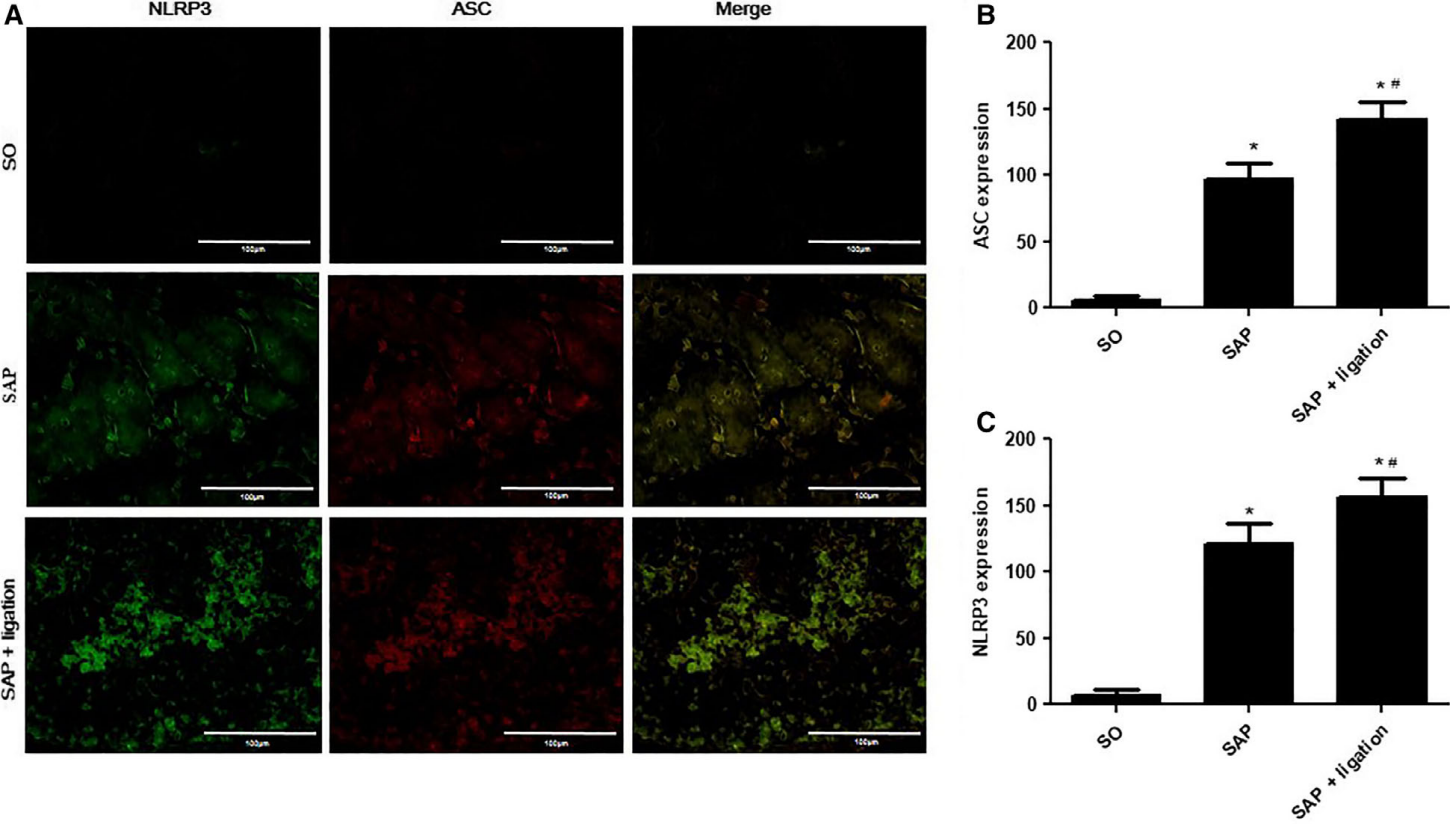
Effects of intestinal lymphatic ligation on the expressions of NLRP3 and ASC in the MLN cells in rats. The representative images of the uptake of NLRP3 and ASC in the intestine and mesenteric lymph nodes cells of rats (A) and summary results of bar graphs for each group (B and C). NLRP3 and ASC were determined by confocal laser scanning microscopy (400× magnification), positive expression of NLRP3 presented as green fluorescence, while positive expression of ASC presented as red fluorescence. The counts of positive expressions of ASC and NLRP3, the fluorescence expressions were counted at least 10 fields for each slide. Scale bars = 100 µm. There were six rats used for each experimental group (*n* = 6) and expressed as mean ± SD. *P* values were derived by LSD (L) test and Dunnett's T3 test. *P < 0.05 vs SO group; ^#^ *P* < 0.05 vs SAP group.

To evaluate the effect of mesenteric lymphatic duct ligation on the nonspecific immune (innate immunity) response of the intestinal tract in the SAP rats, we investigated the effects of mesenteric lymphatic duct ligation on the expressions of NLRP3 and apoptosis‐associated speck‐like protein containing a caspase recruitment domain (ASC) in the sections of small intestines and MLN cells by IF. NLRP3 (green fluorescence) was mainly expressed in the cytoplasm of intestinal epithelial cells and lamina lymphocytes. ASC (red fluorescence) was mainly expressed in the cytoplasm of intestinal epithelial cells and lamina lymphocytes. The expression of ASC was colocalized with NLRP3 expression. In the small intestinal tissues (Fig. 4) and MLN (Fig. 5) of the SO group, the expression of NLRP3 and ASC was very low. However, the SAP group was stained positively for NLRP3 and ASC. Expression in the SAP + ligation group was notably increased compared with the SAP group (*P* < 0.05).

### Expression of NLRP3 in the small intestine by western blotting

**Fig. 6:**
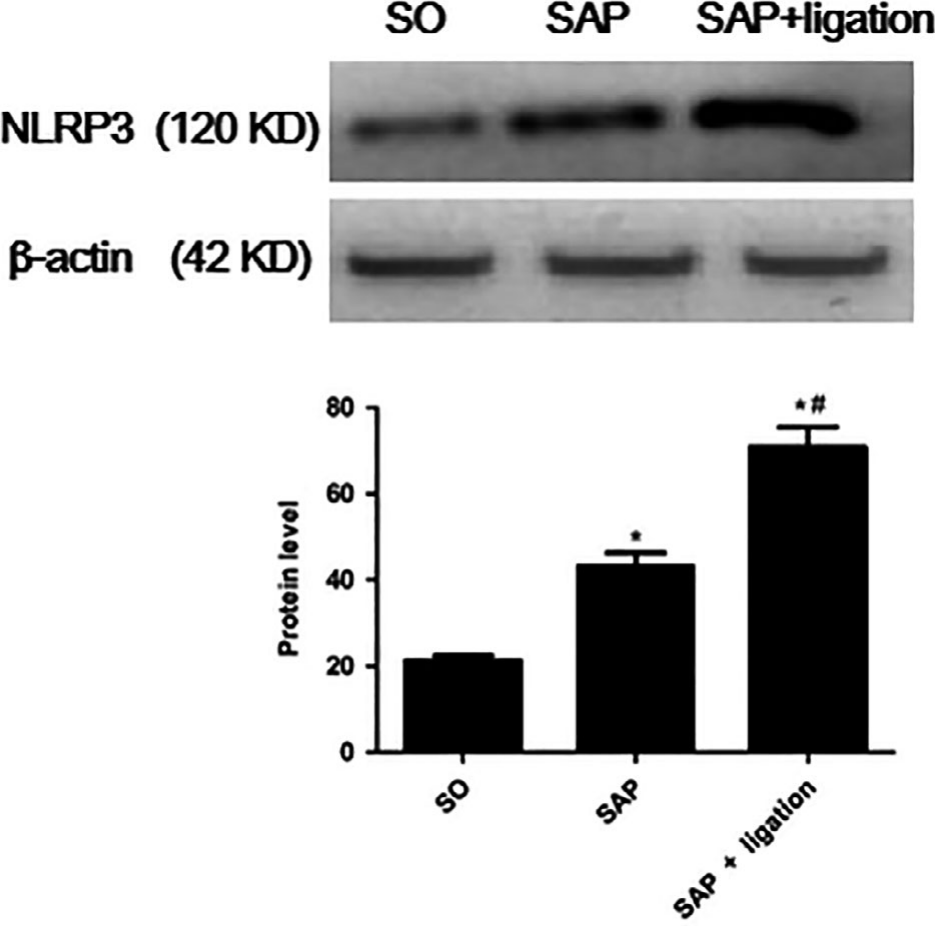
The levels of NLRP3 in the small intestinal tissues by western blotting. There were six rats used for each experimental group (*n* = 6) and expressed as mean ± SD. *P* values were derived by LSD (L) test and Dunnett's T3 test.*P < 0.05 vs SO group; ^#^ *P* < 0.05 vs SAP group.

As was shown in Fig. 6, the expression of NLRP3 in the small intestine in the SAP rats was notably higher than those in the SO rats (*P* < 0.05), but in the SAP + ligation group was higher than that in the SAP group. The results indicated that intestinal lymphatic ligation may aggravate intestinal injury.

### Expression of Caspase‐1 and SIgA with immunohistochemistry

**Fig. 7:**
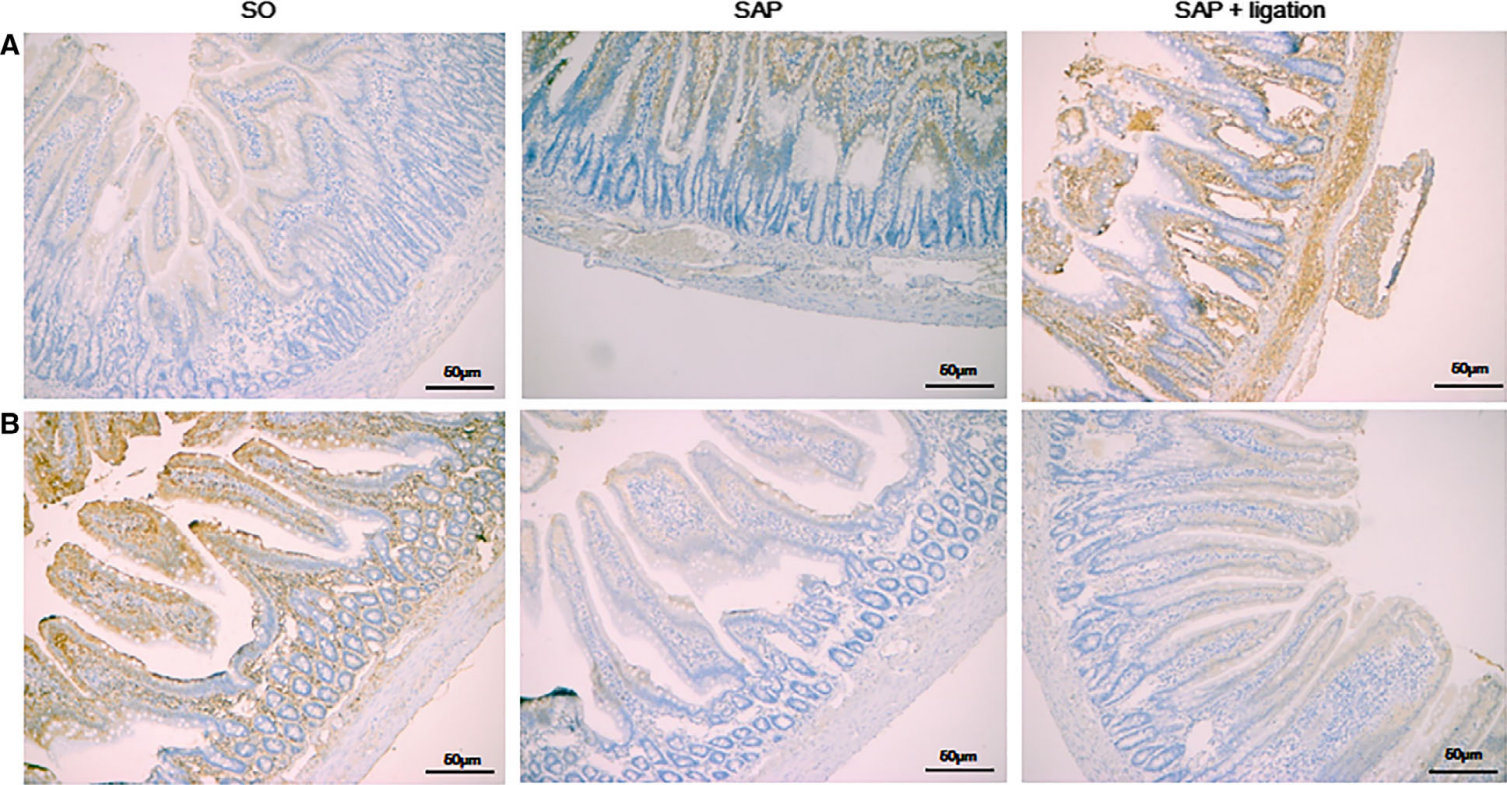
Effects of intestinal lymphatic ligation on the levels of caspase‐1 (A) and SIgA (B) in small intestinal tissues by IHC analysis. Scale bars = 50 µm. There were six rats used for each experimental group (*n* = 6). * *P* < 0.05 vs SO group; # *P* < 0.05 vs SAP group

Caspase‐1 is able to transform active pro‐interleukin‐1β (IL‐1β) to biological active IL‐1β which is a major inflammatory factor. It binds to its receptor after released into extracellular space to induce inflammatory mediators and cause inflammation. Therefore, we evaluated the expression of caspase‐1. Using immunohistochemical methods, it was determined that the expression of caspase‐1 was primarily detected in the intestinal intraepithelial in the cytoplasm. In the intestinal tissue of the SO group, caspase‐1 expression was very low. However, the expression of caspase‐1 in the SAP group was stained positively. Notably, the positive expression of caspase‐1 in the SAP + ligation group increased compared with the SAP group (Fig. 7A). Secretory immunoglobulin A (SIgA) is a key player in humeral immunity and the first line of intestinal mucosal defense. The results showed that the expression of SIgA was markedly decreased in SAP rats compared with that of SO rats. Notably, the positive expression of SIgA in the SAP + ligation group decreased compared with the SAP group (Fig. 7B).

### Ratio of Th1/Th2 in the MLN between different groups

**Fig. 8:**
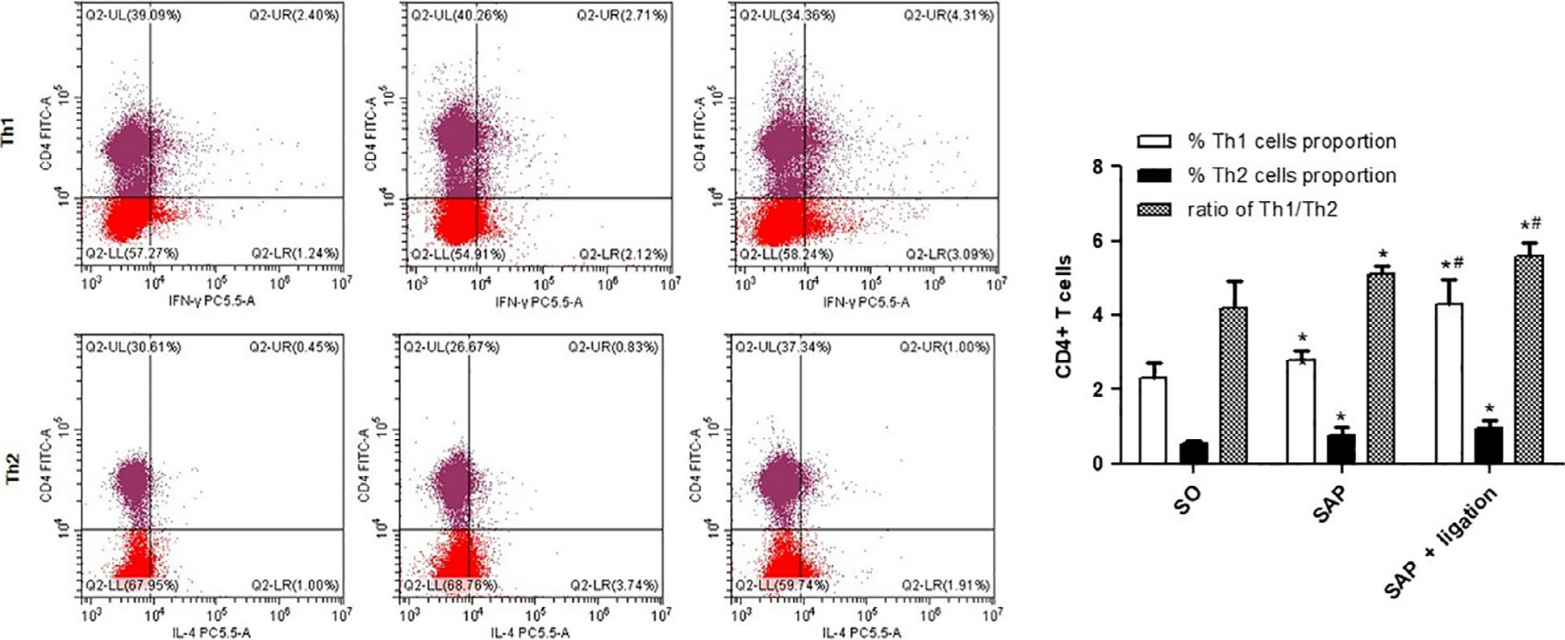
Effect of intestinal lymphatic ligation on the Th1 cells, Th2 cell, and Th1/Th2 ratio by flow cytometry in MLN. There were six rats used for each experimental group (*n* = 6) and expressed as mean ± SD. *P* values were derived by LSD (L) test and Dunnett's T3 test.*P < 0.05 vs SO group; ^#^ *P* < 0.05 vs SAP group.

The expressions of CD4^+^ IFN‐γ^+^ and CD4^+^ IL‐4^+^ present the populations of Th1 and Th2 cells, and the ratio of Th1/Th2 represents their balance. Results showed that the Th1 level in the SAP group was markedly higher than that in the SO group, but significantly lower than that in the SAP + ligation group (*P* < 0.05). The Th2 level in the model group was significantly higher than that in the SO group (*P* < 0.05). Compared with the SO group, the ratio of Th1/Th2 in the SAP group was significantly increased, but significantly lower than in the SAP + ligation group (*P* < 0.05; Fig. 8). That indicated Th1/Th2 was more seriously imbalance in the SAP + ligation group.

### Ratio of Tregs in the MLN between different groups

**Fig. 9:**
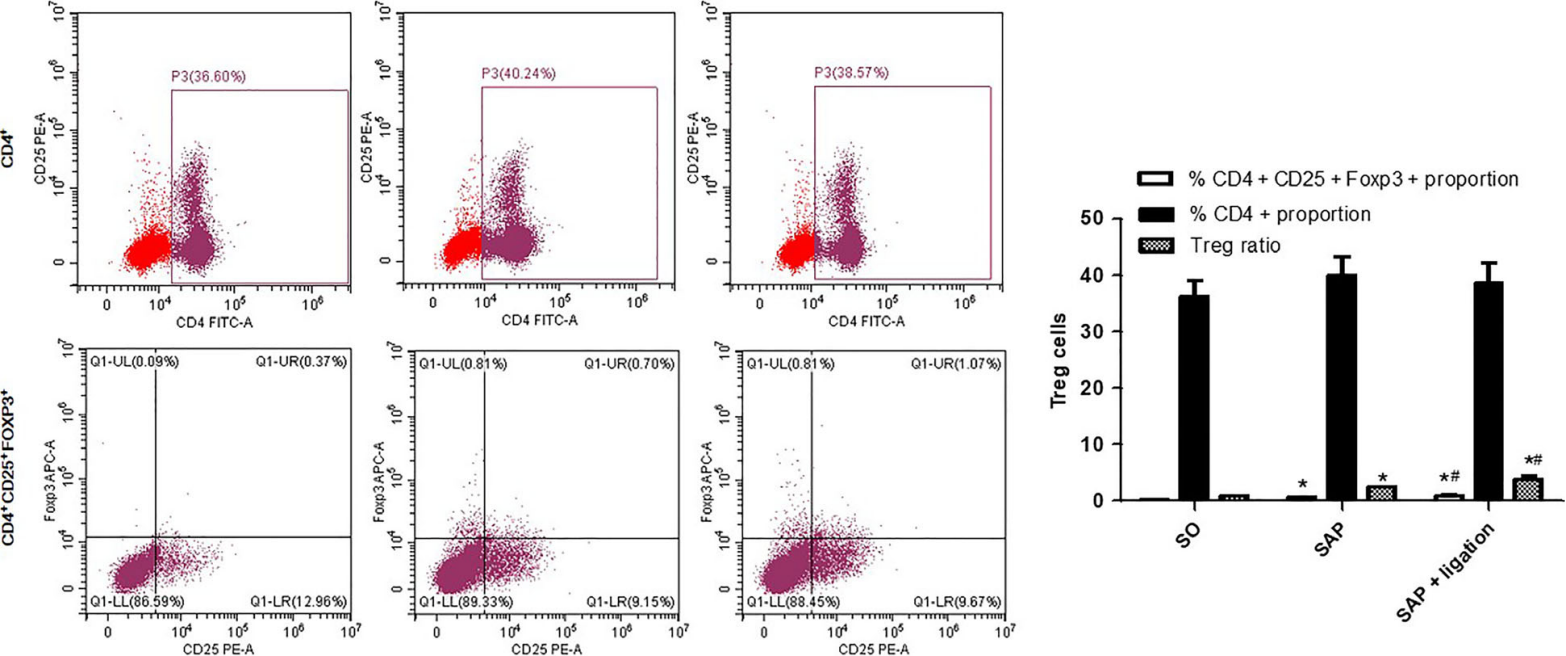
Effect of intestinal lymphatic ligation on the Treg cells in MLN by flow cytometry. There were six rats used for each experimental group (*n* = 6) and expressed as mean ± SD. *P* values were derived by LSD (L) test and Dunnett's T3 test.*P < 0.05 vs SO group; ^#^ *P* < 0.05 vs SAP group.

Tregs have an essential role in maintaining the balance between immune activation and tolerance. The transcription factor CD4^+^ FOXP3^+^ of Tregs is the most specific marker for Tregs. The results showed that the level of CD4^+^ CD25^+^ FOXP3^+^ in the model group was significantly higher than that in the SO group (*P* < 0.05), but was significantly lower than that in the SAP + ligation group (*P* < 0.05). The ratio of Tregs in the SAP group was significantly higher than that in the SO group (*P* < 0.05), but significantly lower than that in the SAP + ligation group (*P* < 0.05; Fig. 9). The data indicated that the population of Tregs was significantly increased in SAP + ligation rats.

## Discussion

The results of the present study indicated that there was no direct protective effect on pancreatic injury with mesenteric lymphatic duct ligation (Fig. 1). Lymphatic ligation may reduce systemic inflammatory response. Ligation of mesenteric lymphatic duct may prevent the intestinal damage factor from entering the blood circulation through the mesenteric lymphatic pathway, and stay in the intestinal tract. The intestinal immune response overactivated due to intestinal lymph ligation in SAP rats. Blocking the intestinal lymph pathway led to a large number of inflammatory cytokines in the intestinal tract, which increased intestinal tract damage (Fig. 2), thus indicating that the intestinal lymphatic pathway was an important pathway involved in the translocation of endotoxin and inflammatory factors.

The intestinal tract, which is the single largest barrier tissue in the human body, has evolved to confine microbiota and resist pathogens, while maintaining the major function of nutrient uptake. It is in direct contact with a large and varied microbial community, and furthermore, the intestinal tract has a large variety of immune cells and structures that help maintain intestinal homeostasis in order to protect against harmful microbes [28]. Researchers have reported that gut barrier function is disordered in SAP and that endotoxemia is associated with SAP [29]. Balanced adaptive immune responses in the intestine are essential for the homeostatic maintenance of the intestinal environment and protection against infection. The role of the intestinal lymphatic system in the natural immune response to inflammatory injury has attracted widespread attention. It is composed of GALT and lymphatic vessels. The former consists of intestinal intraepithelial lymphocytes, lamina propria lymphocytes (LPLs), Peyer's patches, and MLN. During the development of SAP, sensitized T and B lymphocytes translocate from the mesenteric lymph duct and thoracic duct into pulmonary circulation. Researchers have observed that bacterial translocation does not occur via transperitoneal pathways, but most likely via lymphatic spread [30]. Our results showed that intestine damage was more serious in the SAP + ligation group compared with that in the SAP group. It demonstrated that after the ligation of the mesenteric lymph, a large number of cytokines and other immune‐stimulating substances gathered in the gut to induce the immune response, therefore indicating that iIEL regulates functions of the lymphocyte and intestinal epithelial cells by secreting IL‐22, IL‐24, IL‐25, TNF‐α, and TGF‐β. LPLs produce a large amount of SIgA, which can secrete IL‐6 and IL‐5 to promote the differentiation and maturation of IgA B cells to IgA plasma cells.

At present, researchers have demonstrated that intestine damage caused by SAP leads to the release of systemic inflammatory cytokines, including TNF‐α and IL‐1β [31]. TNF‐α and IL‐1β are proposed to play a pivotal role in the pathogenesis of SAP, directly injuring cells and causing necrosis, inflammation, and edema [32]. The present results showed that levels of IL‐1 and TNF‐α in the intestine tissue were increased in SAP + ligation rats when compared with the SAP group (Fig. 3). These findings clarified that ligation of the mesenteric lymph duct promotes the increase in these cytokines by blocking the translocation of inflammatory cytokines via the mesenteric lymph duct. MPO is a biochemical marker for neutrophil infiltration in studies of multiple organ injury in SAP, and its activity is related to the severity of intestine injury [33]. The present results showed that the increase in intestine MPO indicated the progressive aggravation of SAP‐associated intestine injury (Fig. 3). While there were no significant differences in the levels of TNF‐α and MPO between the SAP group and SAP + ligation group. This could be due to the timing of samples taken and experiments. On the other hand, the reduction in systemic inflammation alleviated the intestinal injury due to mesenteric lymphatic ligation to some extent, which indicated that systemic inflammatory reaction was the important reason for serious injury to the body.

Among various inflammasome complexes, the NLRP3 inflammasome is characterized by its role in inducing various human autoinflammatory and autoimmune diseases [34]. It controls the maturation of two pro‐inflammatory IL‐1 family cytokines, IL‐1β and IL‐18, by mediating autoactivation of caspase‐1 [10]. NLRP3 assembly is mediated by protein interaction domains belonging to the death‐fold superfamily, which comprises the death domain, death effector domain, CARD, and pyrin domain (PYD) subfamilies [35]. Immunoglobulin G antibody‐secreting cells (ASCs) are important cell types in the mucosal immune system. A hallmark of inflammasome activation is the ASC speck, consisting of a PYD and a CARD. The oligomerization of ASC creates a number of potential caspase‐1 activation sites, thus leading to inflammasome‐mediated cytokine production [36]. The cysteine protease caspase‐1 plays a critical role in proteolytically maturing and secreting cytokines, such as IL‐1β and IL‐18, and in inducing a pro‐inflammatory programmed cell death mode known as pyroptosis [37]. The present data indicated that the expression levels of NLRP3, ASC, and caspase‐1 were increased in mesenteric lymph duct ligation rats compared with the SAP group (Figs 4, 5, 6 and 7A). The results revealed mesenteric lymph duct ligation enhanced intestinal inflammation through the assembly of NLRP3, ASC, and caspase‐1.

SIgA ASCs are important cell types in the mucosal immune system. SIgA, a dominant Ig in external mucosal secretions, might play a pathogenic role in intestinal inflammatory responses. Substantial evidence has demonstrated that intestinal SIgA regulates the composition and function of the commensal microbiota. SIgA is concentrated in the outer layer of the intestinal mucus along with commensal bacteria to protect the epithelium [38]. In the present study, levels of SIgA were analyzed in the small intestine after ligation of the mesenteric lymph duct; decreased levels of SIgA were found in SAP + ligation rats as a result of intestinal inflammation (Fig. 7B). Our data demonstrated that lymphatic ligation could decrease the secretion of SIgA to promote bacteria into the blood and intestinal damage.

Emerging evidence has suggested that CD4^+^ Th cells can be divided into Th1 and Th2 subsets based upon the cytokines they produce, which are mutually antagonistic. Th1 cell differentiation induced by IFN‐γ and IL‐12 signaling via STAT1 and STAT4 produces reactive oxygen species and mediates innate immunity, respectively, whereas Th2 cell differentiation driven by IL‐4 signaling via STAT6 promotes Ab‐mediated immunity by activating mast and B cells [39]. The balance of Th1/Th2 regulates the immune system under normal conditions. The present data showed that development of intestinal inflammation was regulated by Th1/Th2 bias induced by ligation of the mesenteric lymph duct (Fig. 8).

CD4^+^ CD25^+^ Tregs play a pivotal role in the maintenance of immune homeostasis. Tregs can potentially inhibit microbial clearance or benefit the host by suppressing immune pathology [40]. FOXP3, expressed in CD4^+^ CD25^+^ cells, determines Treg development and function. By inhibiting Gata3 and RORγt to block IL‐4 and IL‐17 expression, FOXP3 plays a critical role in inhibiting both Th2 and Th17 differentiation [41]. Ectopic expression of FOXP3 in CD4^+^ CD25^−^ T cells may endow CD4^+^ CD25^−^ T cells with a Treg‐like suppressive capability to prevent the development of IBD and autoimmune gastritis [42, 43]. In the present study, it was found that levels of CD4^+^ CD25^+^ FOXP3^+^ and Tregs were enhanced significantly in the ligation rats (Fig. 9).

In conclusion, the results demonstrated that ligation of the mesenteric lymph duct can effectively prevent intestinal inflammatory mediators entering the body via the mesenteric lymph duct, and thus, they assembled in the intestine. Consequently, excessive release of inflammatory mediators and excessive immune responses during SAP were induced, thus aggravating intestinal injury.

## Conflict of interest

The authors declare no conflict of interest.

## Author contributions

The manuscript concept was devised by YL, LC, and YX. YL contributed the main draft of the manuscript. YL and LC contributed to the sections on Animal Model. LW and LF contributed to the sections on Immunofluorescence, Immunohistochemistry, and Flow Cytometry. YL and LC contributed to the sections on Statistical analysis. All authors contributed to the writing of the manuscript. All authors read and approved the final manuscript.

## Data Availability

All data generated or analyzed during this study are included in this published article.

## References

[1] Zhong MC and Veillette A (2013) Critical role of SAP in progression and reactivation but not maintenance of T cell‐dependent humoral immunity. Mol Cell Biol 33, 1223–1232. 23319045 10.1128/MCB.01591-12 PMC3592029

[2] Kambhampati S , Park W and Habtezion A (2014) Pharmacologic therapy for acute pancreatitis. World J Gastroenterol 20, 16868–16880. 25493000 10.3748/wjg.v20.i45.16868 PMC4258556

[3] Lu Z , Ding L , Lu Q and Chen YH (2013) Claudins in intestines: distribution and functional significance in health and diseases. Tissue Barriers 1, e24978. 24478939 10.4161/tisb.24978 PMC3879173

[4] McNamee EN and Rivera‐Nieves J (2016) Ectopic tertiary lymphoid tissue in inflammatory bowel disease: protective or provocateur? Front Immunol 7, 308. 27579025 10.3389/fimmu.2016.00308 PMC4985530

[5] Changchien CH , Han YC and Chen HL (2019) Konjac glucomannan polysaccharide and inulin oligosaccharide enhance the colonic mucosal barrier function and modulate gut‐associated lymphoid tissue immunity in C57BL/6J mice. Br J Nutr 123, 319–327. 10.1017/S000711451900285X 31699162

[6] Hosoda N , Nishi M , Nakagawa M , Hiramatsu Y , Hioki K and Yamamoto M (1989) Structural and functional alterations in the gut of parenterally or enterally fed rats. J Surg Res 47, 129–133. 2547111 10.1016/0022-4804(89)90076-0

[7] Rubio CA , Befrits R and Ericsson J (2013) Carcinoma in gut‐associated lymphoid tissue in ulcerative colitis: case report and review of literature. World J Gastrointest Endosc 5, 293–296. 23772267 10.4253/wjge.v5.i6.293 PMC3680619

[8] Moreira CG , Herrera CM , Needham BD , Parker CT , Libby SJ , Fang FC , Trent MS and Sperandio V (2013) Virulence and stress‐related periplasmic protein (VisP) in bacterial/host associations. Proc Natl Acad Sci USA 110, 1470–1475. 23302685 10.1073/pnas.1215416110 PMC3557018

[9] Anderson R and Schmidt R (2010) Clinical biomarkers in sepsis. Front Biosci 2, 504–520. 10.2741/e109 20036897

[10] Luan ZG , Zhang J , Yin XH , Ma XC and Guo RX (2013) Ethyl pyruvate significantly inhibits tumour necrosis factor‐alpha, interleukin‐1beta and high mobility group box 1 releasing and attenuates sodium taurocholate‐induced severe acute pancreatitis associated with acute lung injury. Clin Exp Immunol 172, 417–426. 23600830 10.1111/cei.12062 PMC3646441

[11] Kinnebrew MA and Pamer EG (2012) Innate immune signaling in defense against intestinal microbes. Immunol Rev 245, 113–131. 22168416 10.1111/j.1600-065X.2011.01081.x PMC4624287

[12] Xiong N and Hu S (2015) Regulation of intestinal IgA responses. Cell Mol Life Sci 72, 2645–2655. 25837997 10.1007/s00018-015-1892-4 PMC4479966

[13] Hammer AM , Morris NL , Earley ZM and Choudhry MA (2015) The first line of defense: the effects of alcohol on post‐burn intestinal barrier, immune cells, and microbiome. Alcohol Res 37, 209–222. 26695746 10.35946/arcr.v37.2.06 PMC4590618

[14] Hanning I and Diaz‐Sanchez S (2015) The functionality of the gastrointestinal microbiome in non‐human animals. Microbiome 3, 51. 26552373 10.1186/s40168-015-0113-6 PMC4640220

[15] Kawamoto S , Maruya M , Kato LM , Suda W , Atarashi K , Doi Y , Tsutsui Y , Qin H , Honda K , Okada T *et al*. (2014) Foxp3(+) T cells regulate immunoglobulin a selection and facilitate diversification of bacterial species responsible for immune homeostasis. Immunity 41, 152–165. 25017466 10.1016/j.immuni.2014.05.016

[16] Sun M , He C , Cong Y and Liu Z (2015) Regulatory immune cells in regulation of intestinal inflammatory response to microbiota. Mucosal Immunol 8, 969–978. 26080708 10.1038/mi.2015.49 PMC4540654

[17] Perdigoto AL , Chatenoud L , Bluestone JA and Herold KC (2015) Inducing and administering Tregs to treat human disease. Front Immunol 6, 654. 26834735 10.3389/fimmu.2015.00654 PMC4722090

[18] Xiao J , Wang J , Chen Y , Zhou Z , Gao C and Guo Z (2019) Sauchinone ameliorates intestinal inflammation and promotes Th17 cell production of IL‐10 via Blimp‐1. Biochem Biophys Res Commun 522, 435–441. 31771884 10.1016/j.bbrc.2019.11.122

[19] Clark CE , Hingorani SR , Mick R , Combs C , Tuveson DA and Vonderheide RH (2007) Dynamics of the immune reaction to pancreatic cancer from inception to invasion. Cancer Res 67, 9518–9527. 17909062 10.1158/0008-5472.CAN-07-0175

[20] Carroll IM , Threadgill DW and Threadgill DS (2009) The gastrointestinal microbiome: a malleable, third genome of mammals. Mamm Genome 20, 395–403. 19629594 10.1007/s00335-009-9204-7 PMC4372805

[21] Sairenji T , Collins KL and Evans DV (2017) An update on inflammatory bowel disease. Prim Care 44, 673–692. 29132528 10.1016/j.pop.2017.07.010

[22] Liu YQ , Wang LL , Chen L , Tan XY , Fan L and Xiong YX (2015) Effect of mesenteric lymphatic ligation on liver injury in rats with severe acute pancreatitis. J Pract Med 31, 2252–2254.

[23] Liu YQ (2015) Effect of mesenteric lymphatics ligation on lung injury in Yang Ming Fu Shi rats and intervention of traditional Chinese medicine. Southwest Medical University.

[24] Schmidt J , Rattner DW , Lewandrowski K , Compton CC , Mandavilli U , Knoefel WT and Warshaw AL (1992) A better model of acute pancreatitis for evaluating therapy. Ann Surg 215, 44–56. 1731649 10.1097/00000658-199201000-00007 PMC1242369

[25] Chiu CJ , McArdle AH , Brown R , Scott HJ and Gurd FN (1970) Intestinal mucosal lesion in low‐flow states. I. A morphological, hemodynamic, and metabolic reappraisal. Arch Surg 101, 478–483. 5457245 10.1001/archsurg.1970.01340280030009

[26] Guo W , Ye P , Yu H , Liu Z , Yang P and Hunter N (2014) CD24 activates the NLRP3 inflammasome through c‐Src kinase activity in a model of the lining epithelium of inflamed periodontal tissues. Immun Inflamm Dis 2, 239–253. 25866631 10.1002/iid3.40 PMC4386918

[27] Takahashi N , Saitoh T , Gotoh N , Nitta Y , Alkebsi L , Kasamatsu T , Minato Y , Yokohama A , Tsukamoto N , Handa H *et al*. (2017) The cytokine polymorphisms affecting Th1/Th2 increase the susceptibility to, and severity of, chronic ITP. BMC Immunol 18, 26. 28511637 10.1186/s12865-017-0210-3 PMC5434613

[28] Maynard CL , Elson CO , Hatton RD and Weaver CT (2012) Reciprocal interactions of the intestinal microbiota and immune system. Nature 489, 231–241. 22972296 10.1038/nature11551 PMC4492337

[29] McDermott AJ and Huffnagle GB (2014) The microbiome and regulation of mucosal immunity. Immunology 142, 24–31. 24329495 10.1111/imm.12231 PMC3992044

[30] Kunisawa J , Hashimoto E , Inoue A , Nagasawa R , Suzuki Y , Ishikawa I , Shikata S , Arita M , Aoki J and Kiyono H (2014) Regulation of intestinal IgA responses by dietary palmitic acid and its metabolism. J Immunol 193, 1666–1671. 25031459 10.4049/jimmunol.1302944

[31] Wan YD , Zhu RX , Bian ZZ and Pan XT (2019) Improvement of gut microbiota by inhibition of P38 mitogen‐activated protein kinase (MAPK) signaling pathway in rats with severe acute pancreatitis. Med Sci Monit 25, 4609–4616. 31226101 10.12659/MSM.914538 PMC6599419

[32] Ammori BJ , Fitzgerald P , Hawkey P and McMahon MJ (2003) The early increase in intestinal permeability and systemic endotoxin exposure in patients with severe acute pancreatitis is not associated with systemic bacterial translocation: molecular investigation of microbial DNA in the blood. Pancreas 26, 18–22. 12499912 10.1097/00006676-200301000-00004

[33] Hai W , Ping X , Zhi‐Wen Y and Chun Z (2017) Therapeutic effect and potential mechanism of pioglitazone in rats with severe acute pancreatitis. Braz J Med Biol Res 51, e6812. 29267505 10.1590/1414-431X20176812 PMC5731332

[34] Vaz J , Akbarshahi H and Andersson R (2013) Controversial role of toll‐like receptors in acute pancreatitis. World J Gastroenterol 19, 616–630. 23431068 10.3748/wjg.v19.i5.616 PMC3574587

[35] Lamkanfi M and Dixit VM (2014) Mechanisms and functions of inflammasomes. Cell 157, 1013–1022. 24855941 10.1016/j.cell.2014.04.007

[36] Ying Y , Mao Y and Yao M (2019) NLRP3 inflammasome activation by MicroRNA‐495 promoter methylation may contribute to the progression of acute lung injury. Mol Ther Nucleic Acids 18, 801–814. 31734560 10.1016/j.omtn.2019.08.028 PMC6861628

[37] De Nardo D and Latz E (2011) NLRP3 inflammasomes link inflammation and metabolic disease. Trends Immunol 32, 373–379. 21733753 10.1016/j.it.2011.05.004 PMC3151541

[38] Dick MS , Sborgi L , Ruhl S , Hiller S and Broz P (2016) ASC filament formation serves as a signal amplification mechanism for inflammasomes. Nat Commun 7, 11929. 27329339 10.1038/ncomms11929 PMC4917984

[39] Vajjhala PR , Mirams RE and Hill JM (2012) Multiple binding sites on the pyrin domain of ASC protein allow self‐association and interaction with NLRP3 protein. J Biol Chem 287, 41732–41743. 23066025 10.1074/jbc.M112.381228 PMC3516722

[40] Van Opdenbosch N , Gurung P , Vande WL , Fossoul A , Kanneganti TD and Lamkanfi M (2014) Activation of the NLRP1b inflammasome independently of ASC‐mediated caspase‐1 autoproteolysis and speck formation. Nat Commun 5, 3209. 24492532 10.1038/ncomms4209 PMC3926011

[41] Kaetzel CS (2014) Cooperativity among secretory IgA, the polymeric immunoglobulin receptor, and the gut microbiota promotes host‐microbial mutualism. Immunol Lett 162, 10–21. 24877874 10.1016/j.imlet.2014.05.008 PMC4246051

[42] Marshall NA , Culligan DJ , Johnston PW , Millar C , Barker RN and Vickers MA (2007) CD4(+) T‐cell responses to Epstein‐Barr virus (EBV) latent membrane protein 1 in infectious mononucleosis and EBV‐associated non‐Hodgkin lymphoma: Th1 in active disease but Tr1 in remission. Br J Haematol 139, 81–89. 17854310 10.1111/j.1365-2141.2007.06765.x

[43] Zeng WP , Chang C and Lai JJ (2009) Immune suppressive activity and lack of T helper differentiation are differentially regulated in natural regulatory T cells. J Immunol 183, 3583–3590. 19710452 10.4049/jimmunol.0900146

